# Circulating Soluble Urokinase Plasminogen Activator Receptor as a Predictive Indicator for COVID-19-Associated Acute Kidney Injury and Mortality: Clinical and Bioinformatics Analysis

**DOI:** 10.3390/ijms24087177

**Published:** 2023-04-13

**Authors:** Hidi A. A. Abdellatif, Basma Osman Sultan, Hassnaa M. Nassar, Mostafa Elsaied Elsayed Gomaa, Mohamed Gamal Sakr, Eman Riad, Alhanouf I. Al-Harbi, Jawaher A. Abdulhakim, Manal S. Fawzy, Noha M. Abd El-Fadeal

**Affiliations:** 1 Medical Biochemistry and Molecular Biology Department, Faculty of Medicine, Suez Canal University, Ismailia 41522, Egypt; 2 Oncology Diagnostic Unit, Faculty of Medicine, Suez Canal University, Ismailia 41522, Egypt; 3 Internal Medicine Department-Nephrology Unit, Faculty of Medicine, Suez Canal University, Ismailia 41522, Egypt; 4 Clinical Pathology Department, Faculty of Medicine, Suez Canal University, Ismailia 41522, Egypt; 5 Anesthesia and Intensive Care Department, Faculty of Medicine, Suez Canal University, Ismailia 41522, Egypt; 6 Internal Medicine Department-Tropical Medicine, Faculty of Medicine, Suez Canal University, Ismailia 41522, Egypt; 7 Pulmonology Unit, Internal Medicine Department, Faculty of Medicine, Suez Canal University, Ismailia 41522, Egypt; 8 Department of Medical Laboratory, College of Applied Medical Sciences, Taibah University, Yanbu 46411, Saudi Arabia; 9 Department of Biochemistry, Faculty of Medicine, Northern Border University, Arar 1321, Saudi Arabia; 10 Center of Excellence in Molecular and Cellular Medicine, Faculty of Medicine, Suez Canal University, Ismailia 41522, Egypt

**Keywords:** suPAR, CD87, PLAUR, COVID-19, AKI, mortality

## Abstract

Urokinase receptors regulate the interplay between inflammation, immunity, and blood clotting. The soluble urokinase plasminogen activator system is an immunologic regulator affecting endothelial function and its related receptor; the soluble urokinase plasminogen activator receptor (suPAR) has been reported to impact kidney injury. This work aims to measure serum levels of suPAR in COVID-19 patients and correlate the measurements with variable clinicolaboratory parameters and patient outcomes. In this prospective cohort study, 150 COVID-19 patients and 50 controls were included. The circulating suPAR levels were quantified by Enzyme-linked immunosorbent assay (ELISA). Routine COVID-19 laboratory assessments, including CBC, CRP, LDH, serum creatinine, and estimated glomerular filtration rates, were performed. The need for oxygen therapy, CO-RAD score, and survival rates was assessed. Bioinformatic analysis and molecular docking were run to explore the urokinase receptor structure/function and to characterize molecules as potential anti-suPAR therapeutic targets, respectively. We found higher circulating suPAR levels in COVID-19 patients vs. controls (*p* < 0.001). Circulating suPAR levels positively correlated with COVID-19 severity, the need for O_2_ therapy, the total leukocytes count, and the neutrophils to lymphocyte ratio, while they were negatively correlated with the O_2_ saturation level, albumin, blood calcium, lymphocytic count, and GFR. In addition, the suPAR levels were associated with poor prognostic outcomes such as a high incidence of acute kidney injury (AKI) and mortality rate. Kaplan–Meier curves showed a lower survival rate with higher suPAR levels. The logistic regression analysis confirmed the significant association of suPAR levels with the occurrence of AKI related to COVID-19 and with increased mortality probability within three months of COVID-19 follow-up. Some compounds that can act similarly to uPAR were discovered and tested by molecular docking to identify the possible ligand–protein interactions. In conclusion, higher circulating suPAR levels were associated with COVID-19 severity and could be considered a putative predictor of AKI development and mortality.

## 1. Introduction

There was an international outbreak of a respiratory illness in December 2019. Since then, the World Health Organization has declared a global pandemic known as coronavirus disease 2019 (COVID-19) [1]. More than 118 million COVID-19 cases were reported globally by March 2021 [2]. COVID-19 is characterized by a constellation of symptoms, including high body temperature, weakness, cough, dyspnea, hemoptysis, hypoxemia, widespread lung infiltrates, and consolidation [3].

Growing evidence indicates that severe acute respiratory syndrome coronavirus 2 (SARS-CoV-2) infection causes a hypercoagulable state by contributing to pervasive micro-thrombotic occlusions, endothelial injury, coagulation dysfunction, and impairment of fibrinolysis, and that COVID-19 may be classified as a vascular disorder [4]. Nearly half of hospitalized COVID-19 patients have been associated with acute kidney injury (AKI) [5]. There are no preventative measures to lower AKI incidence, which is strongly associated with poor survival [6].

The soluble urokinase plasminogen activator receptor (suPAR) is a part of the immune system that causes kidney damage. The different ways suPAR binds to ligands are due to its three homologous domains [7]. The uPAR is a glycosylphosphatidylinositol-anchored protein on the cell membranes of different types of cells, including those found in bone marrow, endothelial cells, the immune system, podocytes, and fibroblasts. Phospholipase C cleaves uPAR during times of inflammation and immune activation. Upon activation of immune and endothelial cells, uPAR is released into circulation, where it is converted into suPAR [8].

The inactive zymogen plasminogen must be changed into an active plasmin, which then breaks down cross-linked clots to begin fibrinolysis. The two plasminogen activators: (1) tissue plasminogen activator (tPA) and (2) urokinase plasminogen activator (uPA), are primarily responsible for the liver’s production of plasminogen, which is then converted to plasmin [9]. While uPA can act in open circulation, interaction with the urokinase-type plasminogen activator receptor significantly increases its enzymatic efficiency (uPAR, also called CD87) [10].

Serpins can target both the coagulation and immune responses in viral infections due to the advancement of serine proteases (uPA or tPA) and serine protease inhibitors (serpins) as regulators for thrombosis, thrombolysis, and inflammation. The immune system response to SARS-CoV-2 reveals a different point of view on COVID-19 and fibrinolysis. Numerous inflammatory conditions are linked to the elevated circulating suPAR level [11].

SuPAR is a useful diagnostic and prognostic predictor of severe sepsis, so an elevated suPAR could be used as a therapeutic target for preventing AKI and treating SARS-CoV-2 infection [12]. There are plenty of in vitro and in vivo studies demonstrating that overexpression of suPAR causes severe AKI, which is characterized by the high energy needs and mitochondrial superoxide production [13]. Given suPAR’s involvement in oxidative damage and inflammation, we hypothesized that a high level of suPAR in COVID-19 patients would be associated with a high incidence of AKI. In this sense, this study aimed to explore whether serum suPAR levels can predict the development of AKI or are associated with an increased mortality rate in hospitalized COVID-19 patients.

## 2. Results

### 2.1. Baseline Clinical Characteristics of the Patients and Control Group

The baseline clinical characteristics (Table 1) disclosed a matching of the age and sex between the studied groups. The median age (range) of the control group was 52 (27–83), and for the patients was 55 (20–83). Both sexes were equally distributed in the different groups. About 52% of critically ill patients were intubated, and 40% were on continuous positive airway pressure (CPAP). About 54% of severe cases were on CPAP, and 40% received high oxygen flow therapy, while 88% of the moderate COVID-19 patients were treated using a high oxygen flow therapy.

Most patients in the three COVID-19 groups had very high CO-RAD scores. Meanwhile, most patients in the critically ill group died either soon after hospital admission (36%) or after developing AKI (44%). Nearly 14% of those with moderate COVID-19 infection had evidence of stage 1 AKI, while 12% of severe cases had stage 2 AKI and 6% had stage 3 AKI compared to 15% of stage 2 and 9% of stage 3 AKI in critically ill patients according to the “kidney disease: Improving Global Outcomes; KDIGO” guidelines of AKI classification.

### 2.2. Baseline Laboratory Investigation of COVID-19 Patients Compared to the Control Group

There was a statistically significant difference between groups regarding routine COVID-19 laboratory biomarkers (Table 2) (such as HB, PLT, TLC, neutrophilic and lymphocytic count, ALB, calcium, sodium, potassium, bilirubin (direct and indirect), PT, and PTT), while the serum creatinine admission level showed a normal range average with no statistically significant differences in the patients’ groups compared to the control. Moreover, the mean level of eGFR on admission showed no statistically significant difference, and it was 106.9 ± 16.5 mL/min per 1.73 m^2^ in controls, 108.8 ± 19.6 mL/min per 1.73 m^2^ in moderate cases of COVID-19, 103.6 ± 11.2 mL/min per 1.73 m^2^ in severe cases, and 107.4 ± 17.2 mL/min per 1.73 m^2^ in critically ill patients. Serum inflammatory markers such as LDH and CRP were significantly higher in the different patients’ groups, and the ABG analysis revealed that the O_2_ saturation level had a lower level with the advancement of COVID-19 infection with a mean of 94.9% in moderate, 91.8% in severe, and 87.4% in critically ill patients compared to 97.5% in the control group.

### 2.3. Serum Level of suPAR in Different COVID-19 Groups

**Figure 1:**
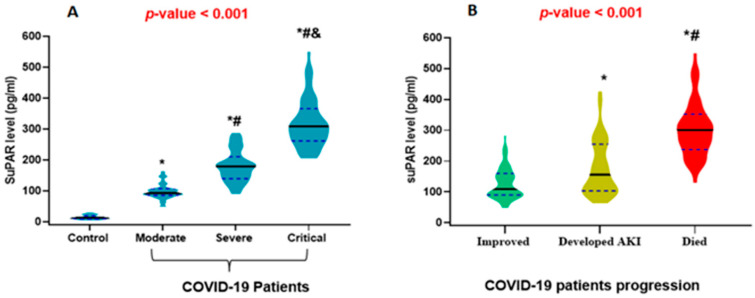
Violin plot demonstrating the median levels of the soluble urokinase plasminogen activator receptor (suPAR): (**A**) COVID-19 patients group compared to control. (**B**) Prognosis of COVID-19. Black lines: indicate the median and blue dots indicate the 25% and 75% quartile. (**Panel A**): * significant compared to control, # significant compared to moderate COVID-19, and & significant compared to severe COVID-19. (**Panel B**): * significant compared to improved cases, # significant compared to those who developed AKI and are still alive. One-way ANOVA and post hoc Bonferroni were used to calculate the *p*-value. Significance was set at *p* < 0.05.

By analyzing the suPAR level in the circulation, the main finding was a significant stepwise rise in the median suPAR level with the advancement of COVID-19 severity compared to the control group, and the median levels were 98.2, 179.3, and 328.4 pg/mL in moderate, severe, and critically ill COVID-19 patients, respectively, compared to 14.33 pg/mL in the control group (Figure 1a). Moreover, in the COVID-19 patients, there was a significantly higher median level of suPAR in those who developed AKI and died either soon after hospitalization or due to AKI progression compared to those who improved with the standard COVID-19 treatment protocol (Figure 1b).

### 2.4. suPAR Level with AKI Stages and Prognosis

**Figure 2:**
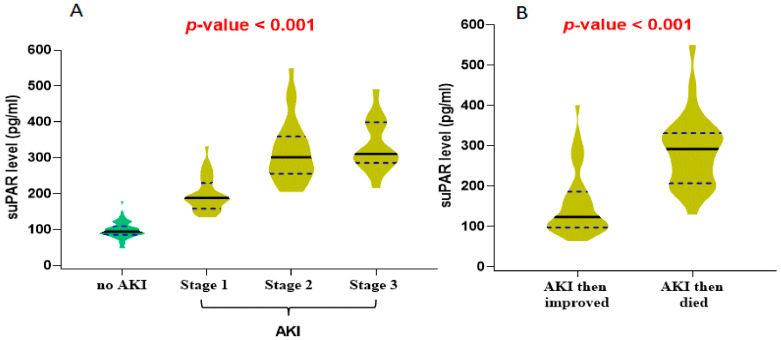
Violin plot for the median level of the soluble urokinase plasminogen activator receptor (suPAR) in COVID-19 patients according to (**A**) AKI stages and (**B**) prognosis of AKI. Black lines: indicate the median and blue dots indicate the 25% and 75% quartile. One-way ANOVA and unpaired t-test were used to calculate the *p*-value. Significance was set at *p* < 0.05.

There was evidence of higher suPAR serum levels with the advancement of AKI (Figure 2a), and the patients that died after developing AKI showed a higher median suPAR level than those who improved from AKI (Figure 2b).

### 2.5. Correlation Matrix for the Interrelationship between suPAR Level and Other Study Variables

**Figure 3:**
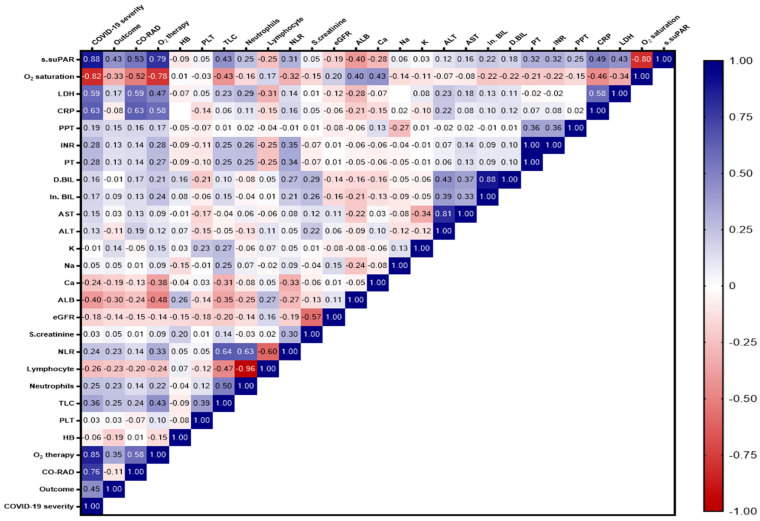
Correlation matrix showing the interrelationship between suPAR level and the different study variables. The blue color indicates a positive correlation, and the red color indicates a negative correlation. The value of r indicates the degree of association. Significance was set at *p* < 0.05.

The matrix showed a significant and strong positive correlation between the suPAR level and COVID-19 severity (r = 0.88) and the need for O_2_ therapy (r = 0.79), and a moderate positive correlation with CRP, LDH, TLC, PT, INR, and NLR (r = 0.49, 0.43, 0.43, 0.32, 0.32, and 0.31), respectively. There was a strong negative correlation between suPAR and O_2_ saturation levels (r = 0.80), a moderate negative correlation between the suPAR level and ALB, calcium, and lymphocyte levels, and a weak negative correlation between the suPAR level and GFR (Figure 3).

### 2.6. Association between suPAR Level and the Significant Clinical and Laboratory Features

**Figure 4:**
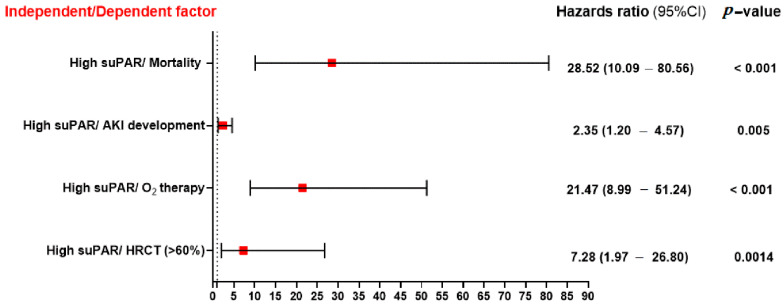
The association between serum suPAR level and other dependent variables such as mortality, AKI development, oxygen therapy, and CT condensation. Significance was set at *p* < 0.05.

Figure 4 shows that patients with a higher median suPAR value were 28.52 times more likely to die from COVID-19 infection progression. They were also 21.47 times more likely to require O_2_ therapy in the form of high oxygen flow, CAPA, or intubation, 7.28 times more likely to have >60% condensations in their chest CT, and 2.35 times more likely to acquire AKI after COVID-19 infection.

### 2.7. Prediction of AKI or Mortality after COVID-19 Infection Using Logistic Regression Analysis

In order to evaluate the biomarkers that were independently associated with AKI or mortality, stepwise binary logistic regression analysis was performed with serum suPAR level, O_2_ saturation, TLC, PLT, PT, PTT, INR, CRP, LD, creatinine, and eGFR laboratory variables, which showed different models with different explanatory variables, and from all models, suPAR was significantly associated with the occurrence of AKI after COVID-19 infection (Table 3) or with increased mortality probability within three-months of COVID-19 infection (Table 4).

### 2.8. Serum suPAR as a Prognostic Marker for COVID-19

**Figure 5:**
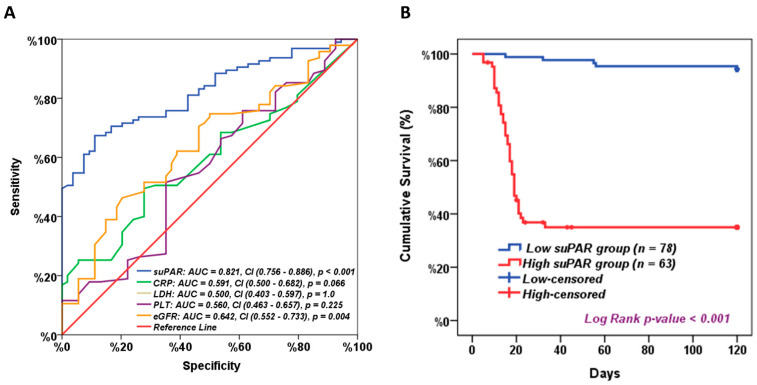
suPAR as a prognostic marker. (**A**) Receiver operating characteristic curve analysis for suPAR level for discriminating patients with bad COVID-19 prognosis from those with good prognosis. AUC: area under the curve. CI: Confidence Interval 95%, and (**B**) Kaplan–Meier survival analysis for all patients based on median suPAR level. *p*-value < 0.05 was considered statistically significant.

ROC analysis showed that the serum suPAR level at a cut-off value of 117.8 pg/mL had a sensitivity of 83.2% and specificity of 53.7%, and the area under the curve (AUC) = 0.821 with a significance of <0.001, compared to the eGFR that had a sensitivity of 74.7% and specificity of 42.6% and the area under the curve (AUC) = 0.642 with a significance of 0.004. The CRP showed a sensitivity of 63.2.7% and specificity of 46.3%, and the area under the curve (AUC) = 0.591 with a *p*-value of 0.066, while the LDH and PLT showed insignificant AUCs that were 0.500 and 0.560, respectively (Figure 5a). For survival analysis, patients were divided into two groups, high versus low suPAR, based on median suPAR cut-off values. Interestingly, Kaplan–Meier curve analysis showed significantly impaired long-term survival for patients with high suPAR levels compared to patients with lower suPAR levels. The median overall survival (OS) was only 21 days for patients with high suPAR levels versus 120 days for low suPAR levels (Figure 5b).

### 2.9. Plasminogen Activator, Urokinase Receptor PLAUR Structural and Functional Analysis

**Figure 6:**
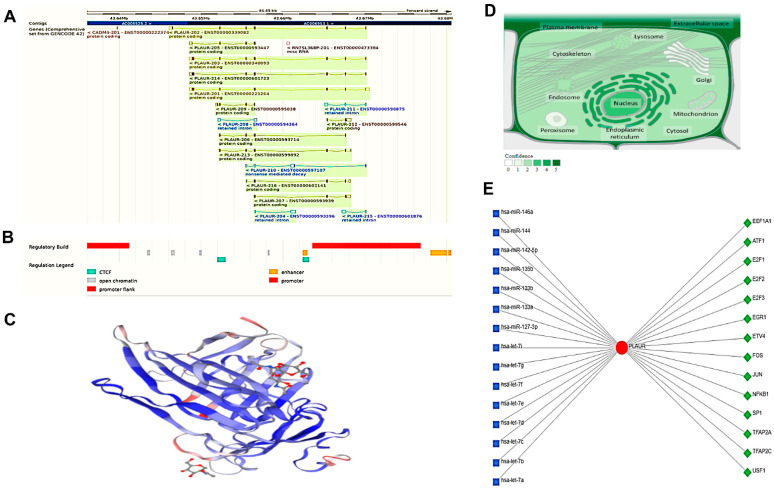
The plasminogen activator, urokinase receptor (PLAUR) structural and functional analysis. (**A**) The chromosomal location and the related transcripts. The blue color is non-coding, while the others are coding transcripts; (**B**) the sites of the promoter and enhancer regulatory factors, either Cis or trans; (**C**) the predicted 3D structure of the PLAUR generated through SWISS modelling; (**D**) the localization of the PLAUR. The color degree is related to protein abundance subcellular, and (**E**) the transcriptional factor (TF)-miRNA coregulatory interactions include 14 TF (green diamonds) interacting with the specified protein and 15 microRNAs (blue squares).

The plasminogen activator, urokinase receptor gene (*PLAUR*; Gene ID: 5329, ENSG00000011422) maps to the long arm of the human chromosome 19 at the locus 19q13.31, spanning the 43,646,095–43,670,547 region on the reverse strand according to the GRCh38.p14 assembly (Figure 6A). This gene has nine exons and is transcribed into 16 different transcripts. Eleven transcripts code for protein, and other transcripts are non-coding transcripts that can be generated through an alternative splicing process and remain as a retained intron or nonsense-mediated decay, respectively (Figure 6A). Moreover, there are some regulatory sequences (cis- and trans-acting) or factors that can regulate the transcription process, as shown in Figure 6B.

The encoded protein was found in the uniport database formed of 335 amino acids in length (36,978 Da), mainly a monomer as shown in the 3D structure (Figure 6C), and it engages in interactions with Sushi Repeat Containing Protein X-Linked 2 and Mannose Receptor C Type 2 (MRC2) (SRPX2). The interaction takes place at the cell surface of the invadopodia membrane and involves the fibroblast activation protein (FAP) (seprase). Additionally, it interacts with Sortilin-related receptor 1 (SORL1) via the N-terminal ectodomain that reduces PLAUR internalization (PubMed:14764453, PubMed:23486467). Additionally, PLAUR-PLAU-SERPINE1 interacts with SORL1 in a ternary complex (PubMed:15053742). The PLAUR protein can be found throughout the cell but is most prevalent in the cell membrane and cellular projection (Figure 6D). 

As PLAUR is a protein that performs vital roles in many biological and cellular processes, the network analysis platform for comprehensive protein expression profiling (Network Analyst version 3.0) was used to recognize the different microRNAs and the transcriptional factors that have a potential impact on PLAUR activity (Figure 6E). Most of these factors and microRNAs were implicated in the inflammatory process.

**Figure 7:**
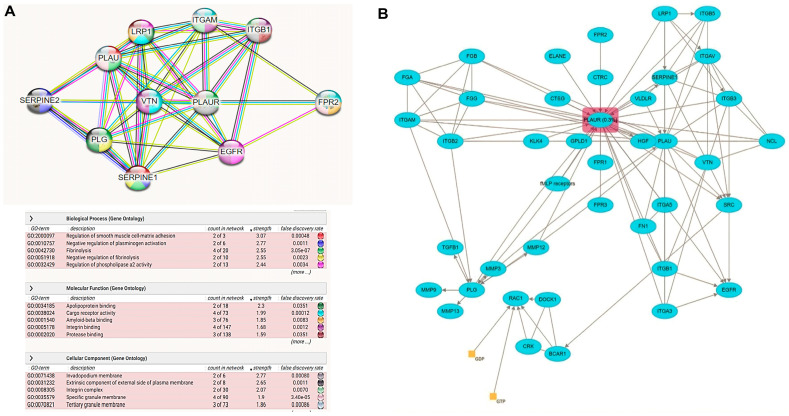
PLAUR pathway analysis: (**A**) The protein–protein interactions network with the top biological processes, molecular functions, and pathways created by the “STRING database”. Each interacting node with the hub PLAUR represents the top 10 predicted functional protein partners with a high confidence level. (**B**) Reactome database fibrinolysis pathway was modified to show how an infection could affect the fibrinolysis process.

The predicted PLAUR protein coexpression network using the default setting of the String database is illustrated in Figure 7A. The type of protein–protein relationships includes (coexpression, genetic interaction, colocalization, shared protein domain(s), and other protein interactions) based on evidence relationships. Among the predicted functional protein partners interacting with PLAUR with a high level of confidence are (1) plasminogen activator inhibitor 1; serine protease inhibitor (SERPINE1) with confidence level = (0.999); it functions as a central control point in the fibrinolysis process regulation. (2) Vitronectin (VTN) with confidence level = (0.999); acts as a cell-to-substrate adhesion molecule, (3) plasminogen (PLG) with confidence level = (0.999); helps in dissolving the fibrin blood clots and acts as a proteolytic factor during inflammation (4) Urokinase-type plasminogen activator (PLAU) with confidence level = (0.999); cleaves plasminogen to form plasmin. (5) Low-density lipoprotein receptor-related protein 1(LRP1) with confidence level = (0.998); involved in the metabolism of complexes between plasminogen activators and their inhibitors. (6) Epidermal growth factor receptor (EGFR) with confidence level = (0.998); activates several signaling cascades to convert extracellular cues into cellular responses. (7) Integrin alpha-M (ITGAM) with confidence level = (0.998); implicated in various adhesive interactions of monocytes, macrophages, and granulocytes as well as in mediating the uptake of complement-coated particles. (8) Formyl peptide receptor-like; N-formyl peptide receptor 2 (FPR2) with confidence level = (0.989); activates the neutrophils. (9) Integrin beta-1 (ITGB1) with confidence level = (0.986); acts as a receptor for collagen. (10) SERPINE2 with confidence level = (0.986); inhibits thrombin (Figure 7A) (data source: https://string-db.org) (last accessed 1 December 2022). Moreover, the Reactome database explored the fibrinolysis pathway, the cell surface PLAUR role in the conversion of plasminogen to plasmin, and the effect of infection or inflammation on dissociated PLAUR from the cell surface of the endothelial cells lining the vessels forming suPAR that leads to a decrease in the fibrinolysis process, and this may explain the coagulation defect that accompanies COVID-19 infection (Figure 7B).

**Figure 8:**
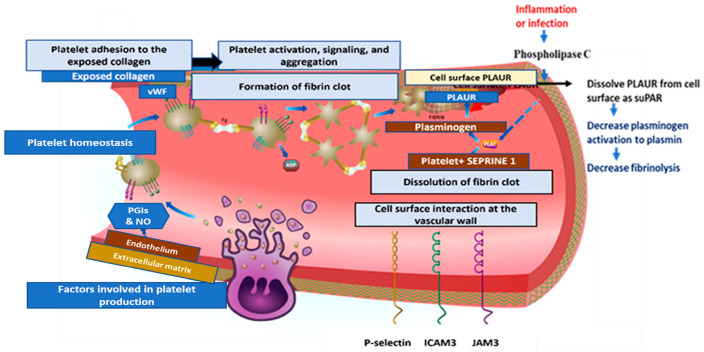
PLAUR targeted pathways generated by the cBioPortal database and Reactome (PB | Hemostasis (reactome.org) (last accessed 3 March 2023).

Finally, the potential target pathway was confirmed by accessing the cBioPortal database, which showed the signaling pathway of the urokinase-type plasminogen activator (uPA) and uPAR. This pathway (R-HSA-109582) is derived from the BioPAX3 of the Pathway Interaction Database (PID) that is curated by NCI/Nature (Figure 8).

### 2.10. Molecular Modeling

Protein and ligands were prepared using Chimera and Moe software. Table 5 shows the tested compounds after adjusting the bond order, adding hydrogens (hidden the non-polar hydrogen), and energy minimization. The first one is the co-crystalized ligand, and the other three compounds are the designed compounds.

### 2.11. UPAR Crystal Structure

**Figure 9:**
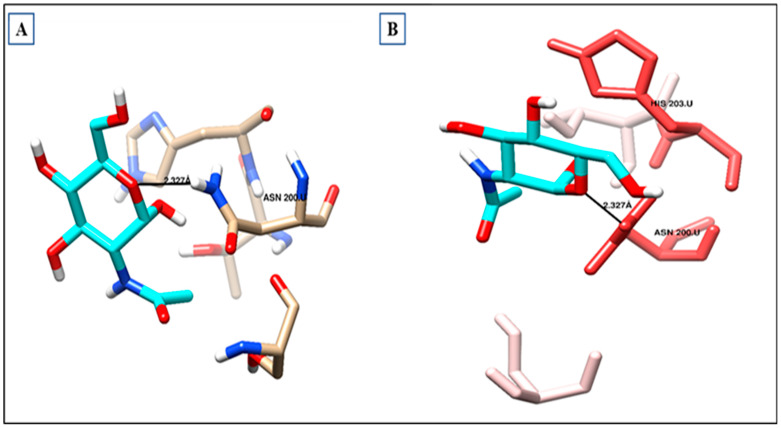
Ligand–receptor interactions: (**A**) binding disposition and (**B**) lipophilic interactions of NDG inside the 2FD6 binding site; red-colored amino acids are the hydrophilic amino acids.

As shown in (Table 6) and (Figure 9a), the co-crystallized ligand NDG creates one hydrogen bond within the binding site pocket of 2FD6 protein through its carbonyl group. There is a hydrogen bond acceptor with the hydrogen of Asparagine200, whose bond length is 2.327 Å (Figure 9b). It does not form lipophilic interactions inside the receptor binding site, but it forms other types of interaction, such as dipole–dipole interaction with Histidine 203 amino acid residue. 

**Figure 10:**
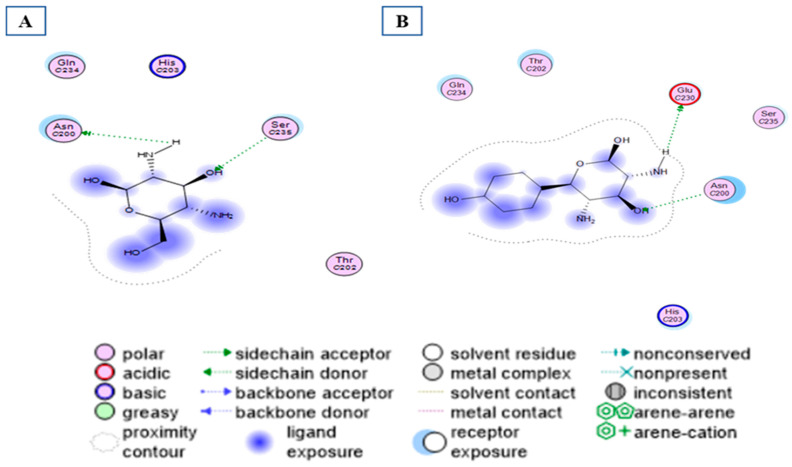
Different interactions of the two tested compounds with the critical amino acids in the receptor site. (**A**) Compound 3 conformer 22, and (**B**) compound 4 conformer 36.

**Figure 11:**
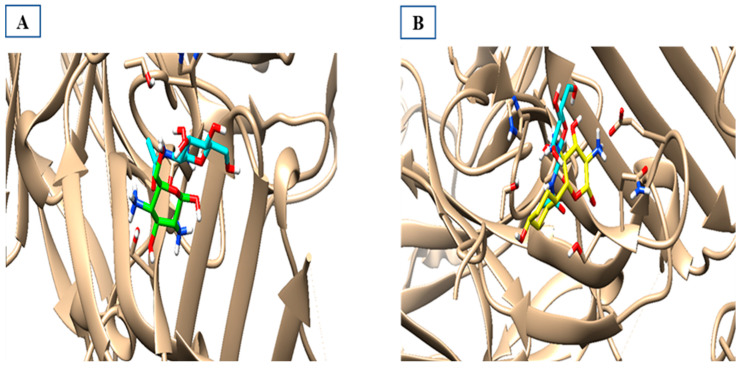
Superimposed models of the docked output to test the most suitable two compounds inside the binding sites of 2FD6. (**A**) The green one is compound 3, the co-crystallized ligand is cyan-colored, and (**B**) the yellow one is compound 4 superimposed with the cyan-colored co-crystallized ligand.

The activity of compounds that can act similarly to UPAR was discovered by performing molecular docking to identify the possible ligand–protein interactions. As shown in (Figure 10a,b), compounds **3** and **4** formed two hydrogen bonds with (ASN200 and SER235) and (GLN230 and ASN200), respectively. After superimposing the two tested compounds, we found that they were docked in the same pocket of the co-crystalized ligand, as shown in (Figure 11a,b).

## 3. Discussion

The urokinase plasminogen activator receptor (uPAR) is a signaling glycoprotein expressed as a GPI-linked receptor on the surface of immune and endothelial cells. It may be cleaved from the cell surface generating soluble uPAR (suPAR) [14]. This latter molecule is implicated in numerous physiological and pathological processes, including chronic systemic inflammation. Moreover, its blood concentrations could reflect the patient’s prognosis and account for the differential outcomes observed among COVID-19-infected individuals [14,15].

Our study revealed a higher suPAR level in COVID-19 cases than in controls, and the level was increased with the advancement of COVID-19 being higher in critically ill patients. This result was consistent with previous studies performed by Rovina et al., Huang et al., and Oulhaj et al., who found that active suPAR was associated with increased COVID-19 severity [12,16,17]. Moreover, in our study, the patients with poor COVID-19 outcomes (developed AKI or died after COVID-19 infection) had a higher baseline suPAR level during hospitalization than patients who improved on the traditional COVID-19 treatment protocol. These data align with Raggam et al.’s study, which found suPAR levels were significantly higher in patients with fatal outcomes than those of survivors from acute diseases, such as systemic inflammatory response syndrome or sepsis [18]. As suPAR was found to be an immune mediator for developing acute and chronic kidney disease, a potential role for suPAR to be used as an essential laboratory parameter in emergency departments was highlighted by Rovina et al. [12].

suPAR can be used to reflect the susceptibility of an individual to develop severe diseases rather than only diagnosing the acute condition, as in the case of COVID-19 or any other infections [19]. The same result was addressed by Chalkias et al., who hypothesized that suPAR plays a major role in the pathogenesis of acute kidney injury by promoting inflammation and reducing oxidative stress [20]. In contrast, Enocsson et al. disagreed with this finding due to the limited number of patients in their study group [21].

The concentration of suPAR in serum was positively correlated with the degree of COVID-19 severity, being higher in severe and critically ill patients. Moreover, there were higher admission levels of suPAR associated with a significantly increased risk of AKI and morbidity after COVID-19 infection. In contrast, the moderate SARS-CoV-2-infected individuals with a lower suPAR admission level had a significantly decreased risk of AKI. Similar outcomes of stratification of suPAR levels concerning COVID-19 disease classification were obtained in a previous study by Enocsson et al., who investigated suPAR in predicting COVID-19 severity and its effect in prolonging the patient’s hospital stay [21].

Given its essential role as a biomarker for inflammation, suPAR is readily detectable in blood samples that can be used easily in the emergency room (ER) or hospital settings [20]. Interestingly, our study explored that suPAR has a better prognostic value (AUC = 0.821) than eGFR (AUC = 0.642), CRP (AUC = 0.591), PLT (AUC = 0.560), and LDH (AUC = 0.500), distinguishing COVID-19 patients who will have poor outcomes due to COVID-19. This result agreed with Azam et al., who reported that patients’ admission suPAR levels could strongly predict AKI development [7]. Moreover, Enocsson et al. discovered that suPAR could be used to predict the length of hospitalization [21]. A study on 57 COVID-19 patients in Greece showed that a suPAR serum level of ≥6 ng/mL was the best predictor for developing severe respiratory failure [12].

We found significantly unfavorable long-term survival for patients with higher suPAR levels than those with lower suPAR levels. Another study by Schultz-Swarthfigure et al. revealed that suPAR level is correlated with a prolonged hospital stay due to other severe conditions with excessive inflammation, such as cardiac surgery, pneumonia in children, and burn injuries [22]. Meanwhile, Rovina et al. [12] suggested suPAR as a potential biomarker to predict mortality risk. In our study, we observed similar findings, with suPAR being the dominant predictor for severe COVID-19-associated complications; however, in the present study, absolute serum suPAR levels were lower than those in Rovain et al.’s study. The exclusion criteria of selection of our study participants may explain this compared to the Greek study cohort and the consequently lower proportion of underlying diseases associated with elevated baseline suPAR levels, as well as the use in the Greek study of a different assay technology to measure suPAR that is more specific and sensitive.

Hayek et al. identified an association between elevated plasma suPAR levels and the development of acute and chronic kidney disease. This association was observed in patients with normal baseline kidney function and independent of conventional risk factors for kidney disease. The current regression model analysis revealed that the suPAR level significantly improved discrimination power to identify the risk of chronic kidney disease, being more significant than that with well-established biomarkers, such as C-reactive protein (CRP). This finding is consistent with the studies of Enocsson et al. and Hayek et al. [21,23] that confirmed the importance of suPAR assessment to predict end-organ damage associated with systemic lupus erythematosus. Furthermore, Kyriazopoulou et al. showed that suPAR is a biomarker that predicts COVID-19 patients’ progression to severe respiratory failure or death. SuPAR is linked to the presence of danger-associated molecular patterns (DAMPs), particularly calprotectin (S100A8/A9) and IL-13, both of which promote inflammation in COVID-19. Calprotectin stimulated plasma monocytes to produce excessive amounts of IL-1. According to the SAVE-MORE (suPAR-guided Anakinra treatment for Validation of the risk and Early Management Of seveRE respiratory failure by COVID-19) phase 3, a double-blind, randomized controlled trial performed by Kyriazopoulou et al., blocking IL-1 with anakinra (an IL-1 receptor antagonist) prevented strong pro-inflammatory responses and resulted in a 70% reduction in severe respiratory failure and a significant reduction in 28-day mortality when compared to the standard of care [24].

Moreover, correlation analysis demonstrated that suPAR levels positively correlate with CRP, neutrophil/leukocyte ratio, and lymphocyte counts. Similarly, Huang et al. demonstrated that the active suPAR is a COVID-19 prognostic biomarker that might assist in the early triage for SARS-CoV-2-infected individuals to prevent virus transmission [16]. Further studies are needed to see whether the elevated suPAR plasma levels in COVID-19 patients are due to the enhanced overexpression of uPAR or due to their increased shedding from the cell surface. 

We observed that COVID-19 patients with higher plasma suPAR levels had unfavorable CRP, LDH, and neutrophil profiles in the correlation analysis. Moreover, Zhao et al. agreed with our results [25]. It has been reported that lymphocyte and monocyte populations decrease, while neutrophil counts increase in COVID-19, and Téllez et al.’s work showed a link between the neutrophil-to-monocyte ratio and COVID-19 in-hospital mortality [26].

Docking, “the process of predicting the conformation of a small molecule when bound to a receptor site”, has been an integral part of drug development for a long time. The ability to perform it effectively and efficiently can have significant implications in the drug discovery process [27]. An in silico virtual screening and molecular docking studies were performed to screen the activity of some uPAR derivatives to be used as a clinical target for uPAR [28]. Thus, we speculated that designing molecules with a similar geometry and affinity to uPAR might ameliorate the worsening of kidney function in the case of COVID-19 infection or future similar epidemics. In this sense, we suggested three molecules that could be essential targets for designing anti-uPAR therapy. We revealed that compounds 3 and 4 had good docking scores, which might explain the expected structure–activity relationship for its mechanism of action as an anti-uPAR agent. This activity suggested its possible impact in decreasing the deleterious effect of high suPAR on kidney function and associated COVID-19 severity. Our finding was consistent with Zhang et al., who examined SERPINE treatment of severe immune-mediated lung damage in SARS-CoV-2 viral infections in mice models and found its effectiveness in preventing disease-mediated damage [29]. It is worth noting that the role of serpins and anti-PLAUR in the fibrinolysis process in COVID-19 is complex, and investigations on the potential therapeutic modulation of these processes with natural, virus-derived, or engineered serpins, expanding the consideration of these proteins beyond only regulation of the fibrinolytic system, may be a valuable pursuit as many of these modulators are already found to be safe and effective, and in some cases, FDA-approved [11]. Designing inhibitors that cross species barriers according to the species-specific residues of uPA and block uPAR–uPA interactions simultaneously would be helpful. More detailed structural information on the interactions of uPAR with other binding partners, such as integrins, is needed to understand its diverse roles in health and disease [30]. Moreover, validation of our results experimentally to minimize the potential docking side effects and to predict the binding kinetics accuracy is warranted before translation into clinics [31].

This study has some potential limitations. SuPAR levels were measured in a small homogeneous study population, with no stratification of cases, making correlation analysis of absolute suPAR levels in a stratified group difficult. In addition, the scope of this research does not allow for an economic cost–benefit analysis of suPAR using highly sensitive and specific chemical assays to validate the cut-off values for the suPAR level. Finally, the prediction of ADME data about Absorption, Distribution, Metabolism, and Excretion of the docked molecules is missing. This later issue will be scheduled to be performed in further research. 

## 4. Materials and Methods

### 4.1. Clinical Analysis

#### 4.1.1. Study Design and Participants

This prospective, observational cohort study included 150 eligible patients with COVID-19 who were admitted to the COVID-19 isolation room of the intensive care unit (ICU) in the “Suez Canal University Hospital, Ismailia, Egypt” from February 2022 to April 2022.

Adult patients (≥18 years) of both sexes who were hospitalized primarily for COVID-19 after being confirmed to be positive via reverse transcriptase-polymerase chain reaction (RT-PCR) assay of an oropharyngeal or nasopharyngeal swab were included. Patients with a prior history of end-stage renal disease (ESRD) or on dialysis, had an autoimmune disease, or had evidence of renal impairment or AKI as a first presenting complaint at admission, had diabetes mellitus, or hypertension were excluded. The patient cohort was divided into three groups (moderate, severe, and critically ill; each = 50). The control group (*n* = 50) matched the patients in age and sex and were confirmed to have negative PCR for COVID-19.

**Figure 12:**
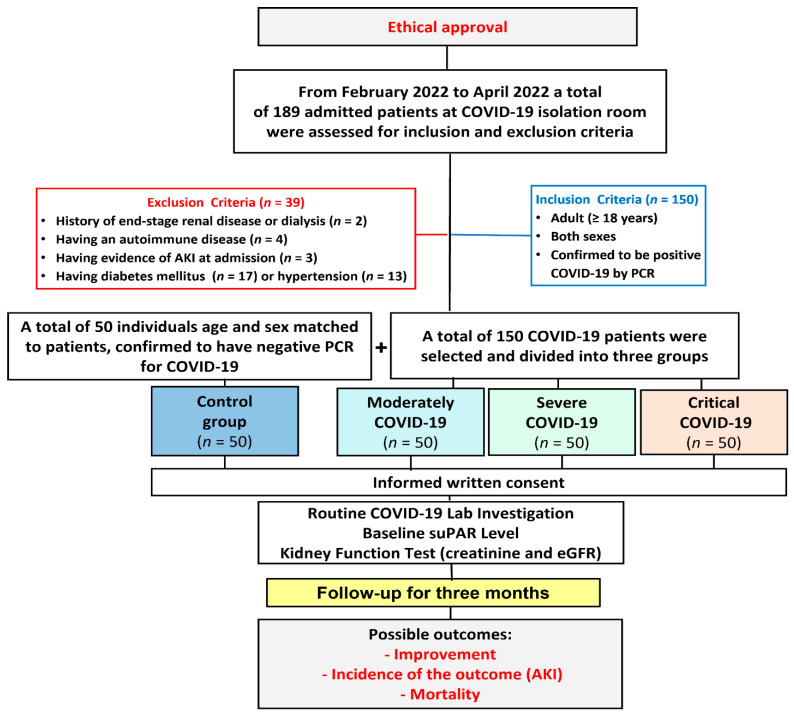
Flowchart of the studied groups and the follow-up until the incidence of the outcomes.

Patients were followed up for three months until the incidence of good outcomes (hospital discharge and improvement) or poor outcomes such as incidence of AKI or transferred to another healthcare facility or death. This was indicated in the flowchart of the work and the follow-up (Figure 12).

#### 4.1.2. History Taking

The data were obtained by reviewing the medical records concerning various factors such as the presence of cardiovascular disease (CVD) (hypertension or congestive heart failure or coronary artery disease), chronic pulmonary disease (asthma or chronic obstructive pulmonary disease or pulmonary fibrosis, or other illnesses affecting the lungs), chronic renal failure, diabetes mellitus, immunosuppression (disease and/or drugs), vaccine received and the number of booster doses, current COVID-19-related medication (anticoagulants, antiviral and/or steroid), and date of confirmed SARS-CoV-2 infection; after patients’ selection, we started to follow up, and during follow-up, the length of hospital stay, hospitalization course, outcomes (AKI incidence or hospital mortality defined by survival status at discharge) were recorded; also, the maximum need for oxygen supplementation (<5 L/min or HFNOT or CPAP or mechanical ventilation), and the need for renal dialysis were recorded.

#### 4.1.3. Sample Collection and Processing

Blood samples were collected in plain and EDTA tubes within 48 h of hospital admission. Plain tubes were centrifuged at 4000 rpm for 15 min to separate serum and transported to the Oncology Diagnostic Unit Lab, Faculty of Medicine, Suez Canal University, to be stored at 80 °C pending further laboratory analysis. EDTA tube samples were used for various hematological parameters’ analysis. 

#### 4.1.4. Baseline Hematological and Biochemical Assessment

Blood samples collected on the EDTA tube (2 mL) were analyzed on a Sysmex XN1000 hematology analyzer (Sysmex Corporation, Kobe, Japan) for hemoglobin and blood cell counts (CBC) as platelets (PLT), total leukocytes count (TLC), monocytes, lymphocytes, neutrophils, eosinophils, basophils. Serum samples separated from the plain tubes (3 mL) were analyzed on Roche Cobas^®^ 6000 autoanalyzer (Roche Diagnostics, Mannheim, Germany) for five parameters (CRP, LDH, sodium, potassium, and creatinine) at the Clinical Chemistry Unit, Suez Canal University hospitals.

The baseline serum creatinine level was measured within 48 h of hospital admission, and from the same sample, the suPAR level was assessed. Serum creatinine measurements were repeated every other day during the hospitalization to follow up the patients. All values were recorded in the patient files. Moreover, the eGFR was calculated using the Chronic Kidney Disease Epidemiology Collaboration (CKD-EPI) equation [32].

#### 4.1.5. Baseline Measurement of Circulating suPAR Level

Commercial ELISA kits from the SunRed biotechnology company assessed plasma suPAR concentrations (Cat. no.2 01-12-5720, China). The kit’s double-antibody sandwich is a reliable method for measuring the concentration of suPAR in samples.

First, 40 uL of serum was added to the precoated well, then 10 uL of suPAR-antibody and 50 uL of streptavidin-HRP, followed by incubation at 37 °C for 60 min. After removing any excess liquid, 50 uL from chromogen solutions (A and B) was added and incubated at 37 °C in the dark for 10 min. Lastly, 50 uL of a stop solution turned the blue color into yellow. The sample’s (suPAR) concentration was positively associated with its color intensity.

The concentration of standards and their 450 nm optical densities (OD) were used to derive a linear regression equation, which was then used to determine the concentration of suPAR by applying the OD values of the standards to the collected samples. The minimum detection level of the applied assay was 5 pg/mL with a sensitivity of 4.368 pg/mL according to supplied vendor data.

#### 4.1.6. Characterization to Determine AKI or COVID-19 Severity

The incidence of AKI during hospitalization was defined based on the use of the Kidney Disease Improving Global Outcomes (KDIGO) standards [33]: AKI stage 1: involves a 1.5–1.9-fold rise in serum creatinine relative to their hospital admission baseline values within seven days or a ≥0.3 mg/dL absolute rise in creatinine over 48 h. Stage 2 AKI: involves a 2.0–2.9-fold rise in serum creatinine compared with their admission levels. Lastly, stage 3 AKI: involves a three-fold rise in serum creatinine compared with their admission values or a rise in creatinine to ≥4 mg/dL or the beginning of renal replacement therapy (RRT).

National Institutes of Health guidelines were used to categorize the severity of COVID-19 in the patient cohort [34] and approximated concerning the highest level of care (pandemic isolation department, intermediate or intensive care unit) and the maximum oxygen need, and the classification was as follows: mild (pandemic department, no oxygen supplementation), moderate (pandemic department, oxygen supplementation <5 L/min), severe (pandemic department or intermediate care unit, oxygen need ≥5 L/min or need CPAP), and critical illness (intensive care unit, with or without mechanical ventilation). 

Because no mild cases were admitted to the hospital, the mild group was not included in this study, while we included other patient groups who presented with moderate, severe, and critical conditions of COVID-19 infection. The definitions of outcomes include the incidence of AKI during hospitalization, defined according to the KDIGO criteria, or the incidence of mortality associated with COVID-19 infection or AKI.

#### 4.1.7. Ethical Considerations

Participants’ rights and privacy during the study were taken very seriously following the guidelines of the Research Ethics Committee at Suez Canal University (reference code for the ethical approval 4663#). The hospital management executive board permission for data collection was obtained at their meeting dated January 2021. Written informed consent was taken from all the participants in the study before the start of the sample and data collection.

#### 4.1.8. Statistical Analysis

Data were subjected to outliers’ detections and normality tests to identify whether the data were parametric or non-parametric using the Shapiro–Wilk normality test at 0.05. Data were described statistically in terms of means and standard deviation or standard error of means. Inferential statistics were carried out using the chi-square test for categorical variables. Unpaired t-test was used to calculate the significance for quantitative variables in two groups, “One-Way Analysis of Variance” for more than two groups or Mann–Whitney U and Kruskal–Wallis tests if the data were non-parametric. A correlation test was employed using Spearman’s correlation coefficient to display a plot as a correlation matrix. The receiver operating characteristic (ROC) curve was utilized to investigate if the suPAR level could discriminate poor outcomes from good outcomes in COVID-19 patients compared to the control. Survival analysis was performed using the Kaplan–Meier test, and the log-rank was applied for its estimation. The *p*-value was set at 0.05 using the computer software Statistical Package for Social Science SPSS (IBM-SPSS ver. 28.0) and GraphPad Prism version 8.0. for Windows, GraphPad Software, San Diego, CA, USA, www.graphpad.com (last accessed 7 April 2023).

### 4.2. In Silico Bioinformatics Analysis

#### 4.2.1. PLAUR Structural and Functional Study

The in silico structural and functional analysis of the *PLAUR* gene and protein was accessed in diverse bioinformatics databases as the National Center of Biotechnology Institute (NCBI) (https://www.ncbi.nlm.nih.gov/gene/5329), gene cards (www.genecards.org), Ensemble (www.ensembl.org), UniProt knowledge base (https://www.uniprot.org/), Compartments to know the subcellular location of PLAUR (https://compartments.jensenlab.org/), “NetworkAnalyst version 3.0” [35] to study the interactive transcriptional factors and microRNAs with PLAUR protein (https://www.networkanalyst.ca); String database version 11.5 [36] was used to explore the protein–protein interactive network with PLAUR gene ontology to enhance the functional enrichment analysis of our target protein (https://string-db.org/); the cBioPortal database platform (https://www.cbioportal.org/datasets) used to define different target pathway related to PLAUR; and finally, the Reactome database was used for pathway enrichment analysis (all databases were last accessed 27 December 2022).

#### 4.2.2. Molecular Modeling 

(a) Molecular optimization of crystallographic protein: using the PDB to obtain the UPAR protein code to help predict an anti-UPAR therapeutic process. 2FD6 code for the UPAR receptor was selected [37]. This PDB code is accessible at (https://www.rcsb.org/structure/2FD6) (last accessed 27 December 2022).

(b) Visualization and chemical analysis of the ligand–receptor interactions by Chimera software version 1.16 [38]: first, UPAR binding site was visualized, the inclination of the original co-crystallized ligand and their primary ligand–receptor interaction in terms of (hydrogen bonding or lipophilic interaction) with the key amino acid residues was studied [39].

#### 4.2.3. Docking Score

The compounds, which were designed using ChemDraw pro 8.0, were tested inside their receptor binding site and analyzed for their main interactions [40]. If a compound forms extra ligand interactions with its receptor binding site compared with the original co-crystallized ligand, it gives the impression that it has a better activity. Generally, the receptor binding sites with ribbon representation and ligands without non-polar hydrogens were highlighted by Cyan color carbon skeleton. In the lipophilic analysis, polar (hydrophilic) amino acid residues were represented in red colors, while the non-polar (lipophilic) ones were represented in blue color [41].

## 5. Conclusions

Serum suPAR levels were significantly related to COVID-19-associated AKI and mortality. Future research could investigate combining serum suPAR with other clinical and laboratory biomarkers to improve risk prediction accuracy. Our findings suggest that suPAR could be used to identify patients who need intensive management and may require a newly designed treatment option. More research into molecular docking is required to determine whether modifying circulating suPAR is a feasible treatment modality.

## Figures and Tables

**Table 1:** Baseline clinical characteristics of studied COVID-19 patients.

Variables		Control	Moderate	Severe	Critical	*p*-Value
		*n* = 50	*n* = 50	*n* = 50	*n* = 50	
		N (%)	N (%)	N (%)	N (%)	
Sex	Male	21 (28%)	24 (32%)	27 (54%)	21 (28%)	0.575
	Female	29 (32.2%)	26 (20.8%)	23 (46%)	29 (23.2%)	
Age	Mean ± SD	53.8 ± 12.1	51.7 ± 13.2	58.2 ± 12.4	52.7 ± 12	0.075
O_2_ therapy	Room air	50 (100%)	6 (12%)	3 (6%)	2 (4%)	<0.001 *
	Low or high O_2_ flow	0 (0%)	44 (88%)	20 (40%)	2 (4%)	
	CPAP	0 (0%)	0 (0%)	27 (54%)	20 (40%)	
	Intubated	0 (0%)	0 (0%)	0 (0%)	26 (52%)	
CO-RADS	No	50 (100%)	(0%)	0 (0%)	3 (6%)	<0.001 *
	High	0 (0%)	6 (12%)	12 (24%)	8 (16%)	
	Very high	0 (0%)	23 (46%)	29 (58%)	29 (58%)	
	PCR	0 (0%)	21 (42%)	9 (18%)	10 (20%)	
Progress	Improved		30 (60%)	23 (46%)	2 (4%)	<0.001 *
	Died soon after admission		0 (0%)	0 (0%)	18 (36%)	
	Developed AKI, then improved		17 (34%)	12 (24%)	4 (8%)	
	Developed AKI and deteriorated		3 (6%)	9 (18%)	4 (8%)	
	Developed AKI and died		0 (0%)	6 (12%)	22 (44%)	
Stage of AKI	No evidence of AKI		30 (60%)	32 (46%)	20 (40%)	0.007 *
	Stage 1		14 (28%)	9 (18%)	6 (12%)	
	Stage 2		6 (12%)	12 (24%)	15 (30%)	
	Stage 3		0 (0%)	6 (12%)	9 (18%)	

Data were presented as frequency and percentage; one-way ANOVA and the chi-square were used to calculate the *p*-values. * Significant at *p* < 0.05. AKI: acute kidney injury, CO-RADS: corona radiology score, CPAP: continuous positive airway pressure, PCR: polymerase chain reaction.

**Table 2:** Baseline laboratory findings of studied COVID-19 patients.

Parameters	Control	Moderate COVID-19	Severe COVID-19	Critical COVID-19	*p*-Value
HB (g/dL)	12.9 ± 2	13 ±1.2	13.17 ± 0.9	12.41 ± 1.7	0.91
PLT (×10^3^/µL)	230.9 ± 71.5	215.3 ± 76.8	239.5 ± 81.7	233.8 ± 102.4	0.519
TLC (×10^3^/µL)	6.9 ± 1.7	7.7 ± 4.2	8.1 ± 3.4	12.8 ± 7.4	0.001 *
Neutrophils (%)	74.5 ± 9.4	76.8 ± 13.5	77.2 ± 12.7	83 ± 10.6	0.001 *
Lymphocyte (%)	19.3 ± 8.2	15.4 ± 10.6	16.2 ± 10.4	11.5 ± 8.5	0.001 *
NLR	7.2 ± 4.1	7.6 ± 5.4	6.6 ± 3.4	13.9 ± 12.6	0.001 *
ALB (g/dL)	3.7 ± 0.3	3.5 ± 0.3	3.4 ± 0.4	3.2 ± 0.5	0.001 *
Calcium (mg/dL)	8.7 ± 0.5	8.7 ± 0.5	8.6 ± 0.5	7.9 ± 1.6	0.001 *
Sodium (mEq/L)	137.1 ± 3.1	136.6 ± 3	136 ± 2.1	138.9 ± 5.7	0.047 *
Potassium (mEq/L)	3.9 ± 0.4	3.6 ± 0.4	3.7 ± 0.5	3.9 ± 0.7	0.078 *
Indirect bilirubin (µg/dL)	0.5 ± 0.2	0.6 ± 0.5	0.5 ± 0.3	0.7 ± 0.3	0.006 *
Direct bilirubin (µg/dL)	0.2 ± 0.09	0.3 ± 0.2	0.2 ± 0.2	0.3 ± 0.3	0.094
PT (seconds)	13.7 ± 1.4	13.5 ± 1.1	14.1 ± 2.2	18.7 ± 9.9	0.001 *
PTT (seconds)	34.4 ± 6.1	35.2 ± 6	38.8 ± 9.4	37.7 ± 8.7	0.015 *
Creatinine (mg/dL)	0.6 ± 0.1	0.7 ± 0.2	0.6 ± 0.1	0.6 ± 0.2	0.213
eGFR (ml/min/1.73 m^2^)	106.6 ± 16.5	108.8 ± 19.6	103.6 ± 11.2	107.3 ± 17.4	0.247
LDH (IU/L)	168.7 ± 22.3	316.1 ± 101.2	355 ± 104.2	348.2 ± 116.7	0.001 *
CRP (mg/dL)	4.9 ± 1.5	89 ± 32.8	74.7 ± 35.7	91.8 ± 49.8	0.001 *
O2 Saturation (%)	97.5 ± 0.9	94.9 ± 1.2	91.8 ± 1.7	87.4 ± 3.9	0.001 *

Data are presented as mean and standard deviation. One-way ANOVA was used to calculate the *p*-values. * Significant at *p* < 0.05. HB: hemoglobin, PLT: platelet, TLC: total leucocytic count, NLR: neutrophils to lymphocyte ratio, ALB: albumin, PT: prothrombin time, PTT: partial thromboplastin time, eGFR: estimated glomerular filtration rate, LDH: lactate dehydrogenase, and CRP: C-reactive protein.

**Table 3:** Binary logistic regression analysis for acute kidney injury (AKI) (dependent variable) and different laboratory markers (explanatory variables).

Model	Variable	B	S.E.	Wald	df	Sig.	Exp(B)	95% C.I. for EXP(B)
#1	suPAR	0.004	0.002	5.944	1.000	0.015	1.004	1.001–1.007
#2	suPAR	0.004	0.002	6.717	1.000	0.010	1.004	1.001–1.008
	CRP	0.009	0.004	3.942	1.000	0.047	0.991	0.983–1.000
#3	suPAR	0.004	0.002	6.613	1.000	0.010	1.004	1.001–1.008
	CRP	0.012	0.005	6.123	1.000	0.013	0.988	0.979–0.998
	LDH	0.004	0.002	4.875	1.000	0.027	1.004	1.000–1.007
#4	suPAR	0.005	0.002	7.512	1.000	0.006	1.005	1.001–1.008
	PLT	0.005	0.002	4.406	1.000	0.036	0.995	0.991–1.000
	CRP	0.015	0.005	8.239	1.000	0.004	0.985	0.975–0.995
	LDH	0.005	0.002	6.040	1.000	0.014	1.005	1.001–1.008
#5	suPAR	0.005	0.002	6.655	1.000	0.010	1.005	1.001–1.008
	PLT	0.006	0.002	6.315	1.000	0.012	0.994	0.989–0.999
	CRP	0.015	0.005	7.489	1.000	0.006	0.985	0.975–0.996
	LDH	0.005	0.002	6.469	1.000	0.011	1.005	1.001–1.008
	eGFR	0.027	0.014	3.558	1.000	0.059	0.974	0.947–1.001

**Table 4:** Binary logistic regression analysis for mortality (dependent variable) during the three months of follow-up and laboratory markers (explanatory variables).

Model	Variable	B	S.E.	Wald	Df	Sig.	Exp(B)	95% C.I. for EXP(B)
#1	suPAR	0.018	0.003	36.030	1	<0.0001	1.018	1.012–1.024
#2	suPAR	0.014	0.003	17.231	1	<0.0001	1.014	1.007–1.021
	O_2_ saturation	−0.180	0.081	4.917	1	0.027	0.835	0.712–0.979

**Table 5:** Two-dimensional and three-dimensional optimized energy-minimized structures of the possible compounds released by using the ChemDraw and Moe programs.

Compound Name and No.	2-D Representation	3-D Representation
2-acetamido-2-deoxy-alpha-d-glucopyranose The co-crystalized ligand Compound No. (1)	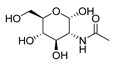	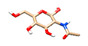
(2*S*,3*R*,4*R*,5*S*,6*R*)-3-amino-6-(hydroxymethyl)tetrahydro-2*H*-pyran-2,4,5-triol Compound No. (2)	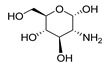	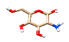
(2*S*,3*R*,4*S*,5*S*,6*S*)-3,5-diamino-6-(hydroxymethyl)tetrahydro-2*H*-pyran-2,4-diol Compound No. (3)	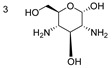	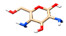
(2*S*,3*R*,4*S*,5*S*)-3,5-diamino-6-(4-hydroxycyclohexyl)tetrahydro-2*H*-pyran-2,4-diol Compound No. (4)	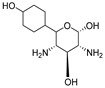	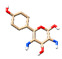

**Table 6:** Summary of the ligand–receptor interactions for the co-crystallized ligand with their receptor binding site.

Molecular target and PDB code	Hydrogen bond analysis			Amino acids involved in the lipophilic analysis	Other bonds
	N.	Hydrogen bond ligand/receptor	Distance(Å)	No lipophilic interaction	Dipole–dipole interaction with His203
Urokinase plasminogen activator receptor (2FD6)	1	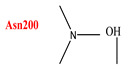	2.327 Å		

## Data Availability

All generated data in this study are included in the article.
